# Acute cardiac injury after subarachnoid haemorrhage: two case reports

**DOI:** 10.1186/1757-1626-2-9293

**Published:** 2009-12-09

**Authors:** Marcello Marcì, Paolino Savatteri, Antonino Pizzuto, Giuseppe Giammona, Baldassare Renda, Francesca Lojacono, Nicola Sanfilippo

**Affiliations:** 1 Cardiology Department, Azienda Ospedaliera Villa Sofia, Palermo, Italy; 2 Neurosurgery Department, Azienda Ospedaliera Villa Sofia, Palermo, Italy

## Abstract

It is well known that cardiopulmonary complications are often associated to subarachnoid haemorrhage. For appropriate therapeutic managing it is very important to distinguish acute coronary syndrome from neurogenic myocardial injury, which is a reversible condition. Furthermore, because the hearts of brain dead patients may be utilized for therapeutic purpose, it has became of importance to rule out erroneous diagnosis of cardiac ischemia in order to avoid rejection of hearts potential suitable for transplantation.

We present a report of two female patients affected by cardiac complications caused by aneurismal subarachnoid haemorrhage admitted to our neurosurgical intensive care department.

## Case presentation

### Case report 1

A 54-year-old Caucasian nun, with history of hypertension, treated with beta-blockers and ACE-inhibitors, was admitted to the neurosurgery department of our hospital for severe nuchal pain, vomit and reduced level of consciousness (Hunt -Hess grade IV). Brain CT scan revealed aneurysm of internal right carotid, in addition subarachnoid haemorrhage (SAH) was evidenced with extension into the III and IV ventricles.

ECG on admission showed normal sinus rhythm, without anomalies of ST-T tract, the corrected QT interval was 455 ms. Laboratory results demonstrated Troponin T level of 3.75 ng/ml (normal value < 0.03 ng/ml), creatinine kinase (CK) was 386 UI/l (normal value < 190 UI/l), NT pro BNP (brain natriuretic peptide) peak level was 259.5 pg/ml (normal value <125 pg/ml). The patient was sedated and intubated.

Transthoracic echocardiography documented wall motion abnormalities, namely akinesis of apex and distal interventricular septum, moreover ejection fraction was about 45%. Inotropic agents, oxygen and Furosemide were administered. Clinical condition suddenly deteriorated after second haemorrhage, occurred soon before surgical correction of aneurysm. Since left ventricular wall motion anomalies persisted after declaration of cerebral death, the heart was considered not suitable for transplantation and only liver and kidneys could be successfully transplanted.

### Case report 2

The second patient was a 71-year-old, Caucasian housewife, with no significant medical history, a part of arterial hypertension, she was not smoker nor diabetic. The patient presented with SAH (Hunt-Hess grade III-IV). First ECG demonstrated normal sinus rhythm, with a heart rate of 96/m, and inverted T waves in V1 → V5, the corrected QT interval was 518 ms. After few hours it was observed the onset of paroxysmal atrial flutter that was successfully treated with Amiodarone (900 mg iv). Troponin T peak level was 0.469 ng/ml, at admission K + was 3.4 mEq/L, which after two days increased to 4.2 mEql/L, Na+ was136 mEq/L, NTpro-BNP reached the peak level of 8166 pg/ml. The echocardiogram demonstrated hypokinesis of distal septum and of apical region, moreover ejection fraction was 48%. Clipping of the anterior left carotid was successfully performed. On the third postoperative day, wall motion abnormalities disappeared; the post-operative course was uneventful and the patient was discharged home after some weeks.

## Discussion

In 1947 Byer et al firstly reported ECG changes in patients with cerebrovascular accidents [1]. Since then a conspicuous number of reports have called attention to cardiovascular abnormalities that frequently characterize the course of SAH [2,3].

ECG changes seen in these patients may be divided in two categories: arrhythmias and repolarisation abnormalities.

Alteration of rhythm and conduction have been detected in about 4% of patients with SAH, the most common arrhythmias are sinus bradycardia and atrial fibrillation/flutter (76% of arrhythmias observed by Frontera et al) [4-6]. Potentially life threatening arrhythmias, such as torsades de pointes and ventricular tachycardia, are exceptional (about 0.4%) and they are usually favoured by electrolytic imbalance and prolongation of QT interval (4,5,6). Anomalies of repolarisation are observed in about 25-75% of patients with SAH, especially in the first three days after admission. Because the repolarisation changes are often similar to those seen in myocardial ischemia and infarction, the interest to this subject has increased to avoid erroneous diagnosis of acute coronary syndrome, that could interfere with a correct therapeutic management [7].

Moreover, approximately 40% of patients with SAH show a modest elevation of Troponin, CPK and CK-MB, although they do not rise to levels observed during acute myocardial infarction. Troponin elevation is correlated more to the degree of brain injury than to severity of cardiac dysfunction evidenced by echocardiography [8,9].

Transient abnormalities of regional wall motion are detected by echocardiography in less than 5% of patients with minimal neurological deficit but in approximately half of patients with poor neurological grade [11,12]. Wall motion anomalies occur predominantly in postemenopausal women and in patients with severe neurological deficit and elevated levels of CK-MB and Troponin I. Specifically, features of echocardiographic abnormalities determined by SAH differ from those observed in myocardial ischemia for their inconsistence with ECGraphic changes [11,12].

Among the proposed pathophysiologic mechanisms underlying ECG changes, ischemic heart disease was excluded by both autopsies and coronary angiographies.

The most plausible pathologic theory remains an autonomic dysregulation caused by a lesion of cortical, hypothalamic and mesencephalic centers controlling the autonomic nervous system [13-15].

As a matter of fact, an elevated concentration of catecholamines was observed in the hearts of animal models of intracranial haemorrhage [14,15]. Furthermore, catecholamine plasmatic levels are markedly elevated in patients with ECG changes than in patients without ECG variations [14,15].

Post mortem examinations of patients who died of SAH demonstrated diffuse small and patchy subendocardial lesions, histologically appearing as myocardial contraction band necrosis [16]. Such myocytes necrosis is usually expression of hypercontracted state caused by cellular calcium overload due to toxic levels of catecholamine [17].

The clinical manifestations of SAH-induced cardiac dysfunction are similar to those observed in other conditions determined by massive release of catecholamine, such as tako-tsubo cardiomyopathy or transient left ventricular ballooning. As a matter of fact this syndrome, which predominates in post-menopausal women, is characterized by reversible wall motion abnormalities, slight elevation in myocardial markers, and transitory ST-T changes, in the absence of obstructive coronary artery disease. Although diagnostic criteria for tako-tsubo cardiomyopathy have initially excluded patients with intracranial bleeding, transient left ventricular apical ballooning could be a complication of SAH [18].

Cardiac involvement induced by SAH has several important clinical implications.

Firstly association of ECG changes, elevations of serum markers of myocardial necrosis and left ventricular dysfunction, may mislead to an erroneous diagnosis of myocardial ischemia with delay in the diagnosis of SAH [17]. Since a rapid diagnosis is crucial for timely operation, and a correct diagnosis of intracranial bleeding can avoid inappropriate treatment with thrombolysis, aspirin or heparin [17], patients who present to emergency department with impairment of consciousness associated to electrocardiographic signs of acute myocardial ischemia, should undergo an urgent CT head scan to exclude intracranial bleeding. Although association between acute myocardial infarction and SAH is very rare [19], coronary arteriography may be necessary when a simultaneous acute coronary syndrome is suspected, especially in male patients with low grade neurologic deficit and extremely elevated and persistent Troponin levels. Although cardiac dysfunction caused by SAH is usually reversible, it may onset dramatically with pulmonary oedema, and it might be indicative of a poor outcome.

Last but not least, patients affected by SAH are generally relatively young and healthy patients, that represent potential numerous organ donors [17,20].

There may be the risk of considering the hearts of irreversibly comatose patients not to be fit for a transplant because they often reveal mild-to-severe left ventricular wall motion abnormalities that, as demonstrated by several studies [20], are generally reversible. For this reason Deibert and co-workers recommend a re-evaluation of heart after brain death declaration when a left ventricular dysfunction has been detected soon after diagnosis of SAH, in order to avoid an inappropriate rejection of donor. Since criteria for selecting a donor hearts are not standardized, several parameters, such as Troponin, BNP and catecholamine concentrations as well as echocardiographic features, should be cautiously considered when evaluating a brain-dead donor.

## Abbreviations

CT: computed tomography; ECG: electrocardiogram; SAH: subarachnoid haemorrhage.

## Consent

Written informed consents were obtained from the second patient and from the first patient's relatives for publication of this case report, a copy of the written consents is available for review by the Editor-in-chief of this journal.

## Competing interests

The authors declare that they have no competing interests.

## Authors' contributions

MM, FL, NS analyzed and interpreted patient's ECG and echocardiograms. MM was a major contributor in writing the manuscript. BR, AP, PS, GG contributed to acquisition of data, analyzed the patient data regarding the neurological disease and the intensive care. All Authors read and approved the final manuscript.
